#  Prognostic Analysis of VEGF, CRP and Contrast-Enhanced Ultrasound Combined with Interventional Embolization for Primary Liver Cancer

**DOI:** 10.34172/aim.34648

**Published:** 2025-10-01

**Authors:** Zheng Dong, Kehui Tong, Yingjie Wu, Mengxiao Lu

**Affiliations:** ^1^Department of General Surgery, The Affiliated People’s Hospital of Ningbo University, Ningbo, China

**Keywords:** Contrast ultrasound, C-reactive protein, Interventional chemotherapy embolization, Primary liver cancer, Vascular endothelial growth factor

## Abstract

**Background:**

To examine the predictive significance of C-reactive protein (CRP), contrast-enhanced ultrasonography (CEUS), and vascular endothelial growth factor (VEGF) in interventional chemoembolization of primary liver cancer.

**Methods:**

A total of 277 patients with primary liver cancer, 162 males and 115 females, aged 41-73 years, were selected from January 2020 to January 2023 in our hospital. These patients received hepatic arterial chemoembolization (TACE). Correlations of VEGF, CRP and contrast-enhanced ultrasound with the progression of TACE within two years were observed. Interventional embolization, comparable preoperative serum VEGF and CRP tests and contrast-enhanced ultrasound (CEUS) quantitative data were used, with the BCLC criteria being stage B, Child‒Pugh grades A‒B, and Eastern Cooperative Oncology Group (ECOG) scores of 0‒1. VEGF was assessed via enzyme-linked immunosorbent assay (ELISA), and CRP was assessed via immunoturbidimetry. Blood was collected at a proximal time point before embolization. CEUS was used to intravenously inject the contrast agent under low mechanical index conditions to obtain dynamic curves of the artery, portal vein and delay period. The ROIs of the lesion and control areas were selected. Two trained radiologists independently measured peak intensity, time to peak, lavage rate and area under the curve in a blinded manner, and the average value was taken for analysis. The primary outcomes were overall survival and progression-free survival, and the secondary outcomes were the objective response rate and disease control rate at 4–8 weeks after surgery. Candidate variable screening was performed via LASSO, a multivariate Cox model was constructed to evaluate prognosis, the proportional hazards hypothesis was tested and processed, and landmark and time-dependent covariate analyses were used for early postoperative indicators.

**Results:**

Contrast-enhanced ultrasound revealed that the maximum tumor tissue strength (IMAX) was 158.74 ± 43.67% and 185.72 ± 51.47% in the progressive and non-progressive groups, respectively. The maximum strength difference between the tumor and parenchyma (IMAX T-P) was 52.18 ± 9.17% (84.52 ± 10.82%), and the tumor tissue ascent times were 8.32 ± 2.85 s and 15.03 ± 6.85 s. The clearance times (WTs) were 12.23 ± 5.14 and 23.05 ± 11.47 s, and the TTP times of the maximum tumor strength were 10.32 ± 3.48 s and 17.05 ± 6.05 s. RT 1, RT t-p, TTP 1, and TTP t-p were not significantly correlated with tumor progression (*P*>0.05). Two groups of patients had conventional VEGF levels [(342.3+/- 72.9, 183.6+/- 62.5 pg /mL] and CRP levels [(19.7+/- 6.8, 11.4+/- 7.3 mg/L], and the difference between before and after comparison [(+/- 33.4, 43.7 to 65.8+/- 71.5) pg /mL, (5.1+/- 4. 2, -3.8 ± 4.0 mg/L], and the difference was statistically significant (*P*<0.05).

**Conclusion:**

The combination of VEGF, CRP and contrast-enhanced ultrasound for the prediction of TACE has potential prognostic application value.

## Introduction

 Primary liver cancer is a common malignant tumor.^1^ According to the annual report of the International Tumor Registry in 2023, the incidence rate of primary liver cancer ranks fourth, and the fatality rate ranks second.^2-4^ It occurs and develops insidiously and is thus difficult to detect in the early stage. Transcatheter arterial chemoembolization (TACE) is a basic integrated arterial treatment administered to patients with Barcelona stage B or middle-stage disease.^5^ It has achieved good outcomes and has become the preferred therapeutic option for patients with Barcelona stage B or middle-stage disease.^6-8^ However, there is also a high recurrence rate after TACE treatment, and the prognostic indicators of TACE are highly important for timely intervention programs and improving the prognosis of patients.^9^ C-reactive protein (CRP) is an acute phase reactant synthesized by the liver and is regulated by pro-inflammatory factors.^10^ It is highly expressed during infection, acute inflammation, cancer, injury, etc. Tumor invasion and metastasis are the pathophysiological basis of recurrence and progression, and the emergence of neovascularization is the key to tumor invasion and metastasis. Vascular endothelial growth factor (VEGF) is overexpressed in the blood of patients with primary liver cancer.^11-13^ These findings suggest that VEGF expression may be a significant predictor in liver cancer patients.^14^ Contrast-enhanced ultrasound can clearly and accurately display the perfusion characteristics of tumor tissue and is widely used in the diagnosis of various tumors, including primary liver cancer.

 We conducted this study to investigate the prognostic value of serum VEGF, CRP and contrast-enhanced ultrasound in patients with hepatocellular carcinoma after TACE.

## Materials and Methods

###  Research Subjects

 We included a total of 162 male and 115 female patients with primary liver cancer, aged 41–73 years, with a median age of 56.5 years, who underwent TACE at our institution between January 2020 and January 2023 were included. Our study strictly adheres to the STROBE guidelines.

###  Inclusion Criteria

 (1) Patients who were diagnosed with primary liver cancer by puncture pathology: Ultrasound-guided percutaneous coarse-needle biopsy (16-18 g) was performed. Two or three tissue samples were routinely obtained for HE and immunohistochemistry (Glypican-3, HeppAR-1, Arginase-1, etc), and were blindly reviewed by two senior pathologists. The results of fine-needle aspiration cytology were not solely used as the basis for enrollment. If only fine-needle aspiration could be performed, typical dynamic imaging features were also considered for enrollment. The imaging diagnostic criteria were supplemented as multiple contrast-enhanced CT/MRI on the basis of liver disease showing high enhancement in the arterial phase and washout in the gate/delayed phase, and it was determined to be LI-RADS 5; (2) Patients who completed TACE, successfully underwent the operation and safely passed the perioperative period; (3) Patients aged > 18 years and who were aware of the research and signed an informed consent form; (4) Minimum follow-up duration: Those without events needed ≥ 6 months of clinical and imaging follow-up before they could be enrolled. Those who showed progression or died within six months were included based on the actual time. Those without events and followed up for less than 6 months were excluded.

###  Exclusion Criteria

 (1) Patients with bacterial infection or dysfunction of important organs: Renal insufficiency was defined as creatinine > 2.0 mg/dL or eGFR < 30 mL/min/1.73 m^2^; Cardiac insufficiency was defined as NYHA III-IV, LVEF < 40%, or acute coronary syndrome in the past 6 months/severe arrhythmia requiring hospitalization. Respiratory insufficiency was defined as indoor air resting SpO_2 _< 90% or PaO_2 _< 60 mm Hg, or COPD with GOLD IV requiring long-term oxygen therapy. Hematopoietic insufficiency was defined as platelet count < 50 × 10^9^/L, absolute neutrophil count < 1.5 × 10^9^/L or hemoglobin count < 8 g/dL. Coagulation disorders were defined as INR > 1.8 (still > 1.5 after correction) or fibrinogen < 1.0 g/L; Severe liver function decompensation was defined as child-Pugh C, refractory ascites, hepatic encephalopathy or total bilirubin > 3 mg/dL, etc; (2) Patients with autoimmune diseases and primary malignant tumors in other parts; (3) Patients with late widespread metastasis; (4) Patients with mental illness or cognitive impairment. This study was conducted in compliance with the requirements of the revised Declaration of Helsinki.

###  Research Methods

 Five milliliters of fasting venous blood was collected from the patient’s median elbow vein 1 day before TACE and 7 days after TACE and placed into an EDTA anticoagulant tube (Beijing Mekmei Biotechnology Development Co., Ltd.). After natural solidification, the samples were centrifuged at 2000 r/min for 15 min (r = 15 cm) at 4 °C and stored at -80 °C. Serum VEGF and CRP levels were detected via enzyme-linked immunosorbent assay. One day before TACE, the patient was subjected to contrast-enhanced ultrasound examination via a GE LOGIQ E9 ultrasonic diagnostic instrument (GE Company, model: LOGIQ-E9), with a frequency of 2–5 MHz. A total of 1.2 mL of SonoV was extracted after it was diluted with 5 mL of normal saline (Bolecco Swiss AG, National Drug Approval number H20110350), and 10 mL was rapidly injected into the superficial vein of the patient’s forearm to observe the angiographic results. The location, size, number, blood flow and other indicators of the intrahepatic lesions were first examined and recorded via 2D ultrasound, and then, the enhancement characteristics of the arterial phase, portal phase and delayed phase were observed and recorded by switching to angiography mode.

 Patients were followed up through outpatient clinics, letters, telephone calls, home visits, WeChat, etc, once a month in the first 3 months and once every 3 months afterwards. According to the plan, outpatient re-examinations were conducted 8 to 12 weeks after the operation, and then every 12 to 16 weeks. Electronic medical records and PACS were retrieved simultaneously. A full-time follow-up officer contacted the patient and the first contact person at least three times by phone, text message or WeChat at different times, recorded the time stamps and filled the unified follow-up form. The outcome (progression/death) was double-verified by imaging reports or death certificates and other materials. The two researchers made a blind determination and resolved any differences through consultation. Loss to follow-up was defined as being unable to obtain any information for at least three consecutive months and having failed to contact three times. CEUS used 2.4 mL of sulfur hexafluoride microbubbles (SonoVue/Lumason) and was intravenously pushed with a 20G indwelling needle at a rate of approximately 1 mL/s. Subsequently, it was rinsed with 5 mL of normal saline at a rate of 1-2 mL/s, with a mechanical index of 0.06-0.08. During the TACE process, the iodized oil chemotherapy emulsion was injected under a selective/superselective microcatheter at a rate of 0.3-0.5 mL/min to strictly prevent reflux until the proximal perfusion was sluggish or substagnant. DEB - TACE microsphere suspension was slowly infused at a rate of 1 mL per 1-2 minutes, and intermittent fluoroscopy was used to assess blood flow.

###  Timing and Mode of Imaging after Contrast

 After the injection, CEUS took t0 as the zero-time point, continuously collected and saved cine (10-15 fps, MI 0.06-0.08) from 0 to 120 seconds, and changed to intermittent scanning between 120-300 seconds, obtaining 5-10 seconds of images every 30 seconds to reduce microbubble destruction. Quantitative analysis generated TIC in continuous segments from 0 to 180 seconds and recorded the peak time calibration. Contrast-enhanced CT/MRI used a multi-phase dynamic protocol (arterial 25-35 seconds, portal 60-70 seconds, with a delay of approximately 180 seconds).

###  Detection of Biochemical Indicators

 VEGF was assessed using a human ELISA kit (R&D Systems, Quantikine, Cat.DVE00), with a detection limit of 9 pg/mL, a linearity of 31.2-2000 pg/mL, repeated Wells, and 4PL fitting. CRP was assessed by immunoturbidimetry (Roche CRP Latex, Cat.05172373190, c702 platform, linear 0.3-350 mg/L).

 VEGF-ELISA was performed using parallel replication Wells of the same batch (n = 2-3), with CV = SD/ mean × 100%. The median indoor variation (intra-assay CV) was 4.6% (IQR 3.2-6.1%), and the inter-laboratory variation was 7.4% (3 days, 3 plates). The indoor CV of CRP immunoturbidimetry (Roche c702) was 1.5% (low value) and 1.2% (high value), and the inter-indoor CV was 2.3% and 2.8%. Preset thresholds: indoor ≤ 10%, inter-indoor ≤ 15%.

###  Parameter Sensitivity Analysis of Risk Prediction Model

 The Cox proportional hazards model was used to evaluate progression-free survival and overall survival. In the presence of competitive risk, the Fine-Gray model was used for sensitivity validation. To enhance the robustness of the prediction, we employed LASSO penalty regression for variable screening and determined the penalty parameters through 10x cross-validation. Considering the time point differences of serum VEGF, CRP and CEUS parameters, we introduced milestone analyses of early post-treatment indicators and time-related covariates on the basis of the baseline model to compare the benefits of the “baseline” and “dynamic” models. Multiple interpolations were performed on the missing data, and the overall analysis followed the TRIPOD reporting specification. The performance of the model was measured by the C-index and the AUC over time, and calibration curves and internal self-service (1000 times) corrections were provided to evaluate overfitting and robustness. The clinical net benefit was quantified through decision curve analysis, and the incremental values of CEUS and serum markers in the combined model were evaluated through NRI/IDI. The results show that in the multivariable framework, the key quantitative parameters of VEGF, CRP and CEUS were all independent predictors.

###  Statistical Methods

 Statistical analysis of the data was performed using SPSS 23.0. To determine the value of each indicator for determining the prognosis of patients, and statistical significance was defined as *P* < 0.05.

## Results

###  Patient Baseline Characteristics

 According to the RECIST 1.1 criteria, the time between first treatment and first evaluation as progression was recorded, and patients were divided into progressive and non-progressive groups on the basis of whether they progressed within 2 years. A total of 152 cases showed progression, including 21 cases within 6 months, 74 cases within 6 to 12 months, and 57 cases within 12 to 24 months. The non-progressive group included 125 patients. The maximum tumor diameter was 2.59 ± 0.68 cm in the progressive group and 2.43 ± 0.72 cm in the non-progressive group. There were statistically significant differences in alpha-fetoprotein (AFP) levels, degree of differentiation (*P* < 0.05, Table 1).

**Table 1:** Comparison of Baseline Characteristics between Progressive and Non-progressive Patients [Cases (%)]

**Clinical features**	**Number of cases**	**Progressive group (n=152)**	**Non-progressive Group (n=125)**	**χ^2^ value**	***P*** ** value**
Gender					
Male	162	92(56.8)	70 (43.2)	0.579	0.447
Female	115	60(52.2)	55 (47.8)		
Age (years)					
< 60	152	86(56.6)	66 (43.4)	0.396	0.529
≥ 60	125	66(52.8)	59 (47.2)		
AFP level (ng/mL)					
≤ 400	83	63(75.9)	20 (24.1)	21.660	< 0.001
> 400	194	89(45.9)	105 (54.1)		
Child-Pugh classification					
A-level	114	58(50.9)	56 (49.1)	1.250	0.264
B-level	163	94(57.7)	69 (42.3)		
Degree of differentiation					
Moderate to low differentiation	203	25(61.6)	78 (38.4)	13.786	< 0.001
Well differentiated	74	27(36.5)	47 (63.5)		
HBSAg					
Positive	191	08(56.5)	83 (43.5)	0.694	0.405
Negative	86	44(51.2)	42 (48.8)		
Number of tumors					
Single	211	13(53.6)	98 (46.4)	0.622	0.430
Multiple	66	39(59.1)	27 (40.9)		
Vascular invasion					
Yes	81	49(60.5)	32 (39.5)	1.460	0.227
No	196	03(52.6)	93 (47.4)		
Capsule integrity					
Complete	38	7 (18.4)	31 (81.6)	13.634	< 0.001
Incomplete	239	45 (60.7)	94 (39.3)		

###  Correlations of pre-TACE Contrast-Enhanced Ultrasound Parameters with Tumor Progression between the Progressive Group and the Non-progressive Group

 The maximum tumor tissue strength (IMAX t), maximum difference between tumor and parenchymal tissue strength (IMAX T-P), tumor tissue rise time (RT t), clearance time (WT), and maximum tumor tissue peak time (TTP t) in the non-progressive group were significantly greater than those in the progressive group (*P* < 0.05). The time of liver parenchymal rise (RT l), the time difference between tumor tissue and liver parenchymal rise (RT t-p), the time difference between the maximum strength of liver parenchymal peak (TTP t-p), and the time difference between tumor tissue and liver parenchymal peak strength (TTP t-p) were not significantly correlated with tumor progression (*P* > 0.05, Table 2).

**Table 2:** Correlation between Pre TACE Contrast-enhanced Ultrasound Parameters and Tumor Progression

**Ultrasound contrast parameters**	**Progress Group**	** Non progressive group**	**T values**	***P*** ** value**
IMAX (%)	158.74 ± 43.67	185.72 ± 51.47	2.608	0.012
IMAX (%)	52.18 ± 9.17	84.52 ± 10.82	5.012	< 0.001
RT(s)	19.24 ± 10.14	21.82 ± 11.47	0.844	0.568
RT(s)	8.32 ± 2.85	15.03 ± 6.85	3.352	0.001
RT(s)	-9.42 ± 8.05	-8.14 ± 7.43	1.388	0.172
WT(s)	12.23 ± 5.14	23.05 ± 11.47	2.974	0.004
TTP(s)	24.85 ± 13.49	26.73 ± 11.52	1.267	0.213
TTP(s)	10.32 ± 3.48	17.05 ± 6.05	2.634	0.009
TTP(s)	-13.48 ± 11.46	-10.85 ± 7.05	1.295	0.203

###  Comparison of VEGF and CRP Levels between the Progressive Group and the Non-progressive Group

 Compared with those of control group patients, the level of VEGF and CRP levels was not significantly different (*P* > 0.05). VEGF and CRP levels before and after treatment were significantly different from those of control group patients (*P* < 0.05, Table 3, Table 4).

**Table 3:** Comparison of VEGF Levels between Progressive and Non-progressive Patients (pg /mL).

**Group**	**Number of cases**	**Before TACE**	**After TACE**	**Difference before and after TACE**
Progress Group	152	282.5 ± 83.7	342.3 ± 72.9	43.7 ± 33.4
Non progressive group	22	275.3 ± 78.1	183.6 ± 62.5	-65.8 ± 71.5
t		0.315	3.412	6.014
*P*		0.776	0.001	< 0.001

**Table 4:** Comparison of CRP Levels between Progressive and Non-Progressive Patients (mg/L)

**Group**	**Before TACE**	**After TACE**	**Difference before and after TACE**
Progress group	15.6 ± 8.2	19.7 ± 6.8	5.1 ± 4.2
Non progressive group	14.7 ± 9.3	11.4 ± 7.3	-3.8 ± 4.0
t	0.394	2.763	3.217
P	0.683	0.008	0.002

###  Potential Prognostic Value of VEGF and CRP Combined with Contrast-enhanced Ultrasound in Predicting Tumor Progression

 Using 2-year progression as the gold standard, the ROCs of VEGF before and after TACE, CRP before and after TACE and TTP were plotted. The AUCs of VEGF and CRP were greater than that of the TTP in predicting tumor progression (Table 5).

**Table 5:** Potential Prognostic Value of VEGF, CRP Combined with Contrast-enhanced Ultrasound in Predicting Tumor Progression

**Index**	**Sensitivity**	**Specificity**	**AUC**	**95% CI**	**Cutoff**
VEGF	0.961	0.787	0.927	0.890～0.955	10.2
CRP	0.862	0.844	0.932	0.895～0.959	0.13
TTP	0.895	0.697	0.829	0.779～0.872	12.3
VEGF, CRP or TTP	0.993	0.713	0.853	0.822～0.905	
VEGF, CRP and TTP	0.776	0.959	0.868	0.806～0.893	

**Figure 1:**
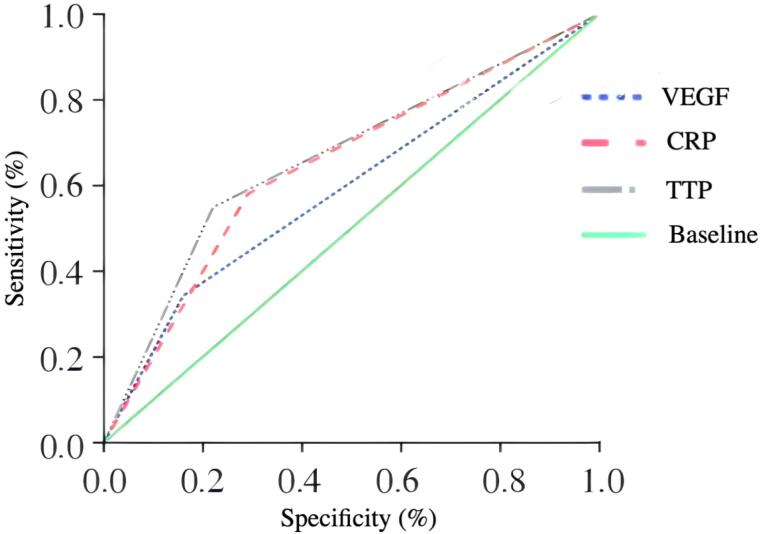
ROC Curve Predictive Analysis

 The predictive ability of TTP of VEGF, CRP and CEUS for overall survival was evaluated by time-dependent ROC. The results showed that all three were significant. Among them, the curve of TTP at each follow-up time point was generally higher than that of CRP and VEGF, and its discriminative performance was the most stable. The discrimination of TTP in medium and long-term follow-up was more prominent than in short-term follow-up. The performance of CRP as a single indicator was relatively poor, but when combined with other indicators, it showed a significant value. Further risk stratification was conducted based on the optimal cut-off point. The poor data of each indicator all pointed to a poor prognosis, which was consistent with the trend shown by time-dependent ROC, supporting its role as an effective tool for prognosis assessment and emphasizing the clinical value of the combination of multiple indicators (Figure 1).

## Discussion

 Eighty percent of the blood supply of the normal liver tissue comes from the portal vein, whereas 90% of the blood supply of the liver cancer tissue comes from the hepatic artery.^15-17^ Hepatic artery embolization can effectively reduce the blood supply of the cancer tissue, causing the tumor to lose its main blood supply, becoming partially or completely necrotic, and prolonging the action time of chemotherapy drugs.^18-20^ To reduce drug release in the body and drug distribution in normal tissues and reduce adverse reactions, the comprehensive treatment mode based on TACE has gradually become the standard treatment for liver cancer, which has lost the opportunity for surgery.^21^ However, collateral circulation or neovascularization can easily be established in the lesion after TACE treatment, resulting in a survival rate of 24%~63% of patients within 2 years after surgery.^22^

 In this study, there were differences in AFP level, degree of differentiation and capsule integrity between patients in the progressive group and those in the non-progressive group, which was basically consistent with the findings of previous studies.^23-25^ There was no significant difference in the progression rate between patients with a single lesion and those with multiple lesions. New indicators should be sought to predict the prognosis of patients with multiple hepatocellular carcinomas.^26^ There were differences in CRP and VEGF levels between patients in the progressive and non-progressive groups after TACE, and the increase in the CRP level was considered to be related to the increase in the levels of pro-inflammatory factors stimulating tumor cell necrosis after TACE.^27^ Patients with increased CRP levels after TACE have a higher rate of progression, which is considered to be related to the growth of tumors stimulated by pro-inflammatory factors.^28-30^ VEGF is the strongest vascular growth factor involved in all aspects of tumor vascular growth and is highly correlated with tumor growth, invasion and metastasis. Increased VEGF levels after TACE are associated with poor prognosis after TACE in patients with liver cancer, and the results of this study are consistent with these findings, suggesting that the overexpression of VEGF after TACE may be an important predictor of residual tumor in patients with liver cancer.^31^ A blood index combined with imaging is helpful for more accurate prognostic assessment of liver cancer patients after TACE.^32^ CEUS can be used to observe tumor microcirculation information in real time; a time‒signal intensity curve can be used to analyze tumor invasion, metastasis trends and tumor vascularization quantitatively.^33-35^ Changes in VEGF and CRP levels before and after TACE and the results of preoperative CEUS were used to evaluate patient prognosis after TACE.^36-38^ The results revealed that the preoperative diagnostic AUC of the TTP was lower than the differences in VEGF and CRP, but the sensitivity of positive results for one of the three was as high as 0.993, and the specificity of positive results for all three was 0.959. The combination of these three methods can help predict patient prognosis after TACE surgery.^39^ Patients with one of the three positive methods have a greater risk of progression, and patients with all three positive methods are almost certain to progress.^40^

 VEGF, CRP combined with CEUS perfusion parameters can be used for prognostic stratification after TACE: For high-risk patients (with the highest quartile in the total score of the chromatogram), it is recommended to shorten the image interval to 8 weeks, conduct early re-evaluation, and consider intensive/systemic treatment. Low-risk cases can be followed up as usual. The model can also be used before surgery to identify those who are not suitable for embolization alone, and for dynamic monitoring after surgery to guide the timing of re-TACE. A nomogram and an online calculator are provided for trial use as decision-making assistance, which should be combined with BCLC, liver function and MDT assessment.^41^

 This study was a single-center retrospective study, and there may be selection bias and residual confounding (such as insufficient measurement of antiviral medication, postoperative additional treatment, etc), as well as extrapolation limitations. CEUS quantification is operator- and equipment-dependent. Despite unified protocols, blind double evaluation and ICC assessment, it is still difficult to completely avoid measurement errors. Some images may be biased due to quality control and exclusion. Only internal self-correction was implemented, lacking external validation and prospective impact studies, and no cost-effectiveness assessment was conducted. When some variables are missing, multiple imputation is adopted. The amount of early events is limited, which may reduce the stability of effect estimation. No central imaging/pathological review was conducted, the follow-up intervals were heterogeneous, and the TACE regimens were also different. A prospective multi-center external validation (≥ 10 centers, unified CEUS protocol, central review) will be carried out, with a pre-registration plan and a target of ≥ 300 PFS events. The primary endpoint was PFS at 12/24 months, and the secondary endpoint was OS. Internal and external cross-validation is adopted to evaluate discrimination and calibration.

## Conclusion

 In this study, a prognostic model was constructed by integrating VEGF, CRP and CEUS under the background of interventional embolization, which was superior to the baseline model that only used stage, liver function, tumor burden, AFP for discrimination and calibration, and brought net benefits to decision-making. This model can be used for risk stratification and follow-up, as well as for optimizing the timing of re-ACE and the transition to systemic treatment.
